# *Drosophila melanogaster* Transcriptome Response to Different *Wolbachia* Strains

**DOI:** 10.3390/ijms242417411

**Published:** 2023-12-12

**Authors:** Nataly E. Gruntenko, Maksim A. Deryuzhenko, Olga V. Andreenkova, Olga D. Shishkina, Margarita A. Bobrovskikh, Natalja V. Shatskaya, Gennady V. Vasiliev

**Affiliations:** Institute of Cytology and Genetics SB RAS, 630090 Novosibirsk, Russia; maksimd@bionet.nsc.ru (M.A.D.); andreenk@bionet.nsc.ru (O.V.A.); shishkinaod@bionet.nsc.ru (O.D.S.); eremina@bionet.nsc.ru (M.A.B.); shatskaya@bionet.nsc.ru (N.V.S.); genn@bionet.nsc.ru (G.V.V.)

**Keywords:** *Wolbachia*, wMelCS, wMelPlus, *Drosophila melanogaster*, transcriptome, DEGs, *Turandot*, *CrzR*, alkaline phosphatase

## Abstract

*Wolbachia* is a maternally inherited, intercellular bacterial symbiont of insects and some other invertebrates. Here, we investigated the effect of two different *Wolbachia* strains, differing in a large chromosomal inversion, on the differential expression of genes in *D. melanogaster* females. We revealed significant changes in the transcriptome of the infected flies compared to the uninfected ones, as well as in the transcriptome of flies infected with the *Wolbachia* strain, wMelPlus, compared to flies infected with the wMelCS^112^ strain. We linked differentially expressed genes (DEGs) from two pairwise comparisons, “uninfected—wMelPlus-infected” and “uninfected—wMelCS^112^-infected”, into two gene networks, in which the following functional groups were designated: “Proteolysis”, “Carbohydrate transport and metabolism”, “Oxidation–reduction process”, “Embryogenesis”, “Transmembrane transport”, “Response to stress” and “Alkaline phosphatases”. Our data emphasized similarities and differences between infections by different strains under study: a wMelPlus infection results in more than double the number of upregulated DEGs and half the number of downregulated DEGs compared to a wMelCS^112^ infection. Thus, we demonstrated that *Wolbachia* made a significant contribution to differential expression of host genes and that the bacterial genotype plays a vital role in establishing the character of this contribution.

## 1. Introduction

*Wolbachia* are widely perceived as an insect parasite since it is found in up to 40–60% of arthropod species [1,2]. However, despite the strong negative influence on the hosts shown for certain *Wolbachia* strains [3,4,5,6], insects have to coexist with this bacterium. It seems that there is a more ambiguous relationship between *Wolbachia* and their host. In some (but not all) host species, a *Wolbachia* infection causes a harmful outcome through four main mechanisms: cytoplasmic incompatibility (CI), male killing, feminization and parthenogenesis [7]. This might result in reduced fertility, among other things. At first glance, it is not obvious how reduced fertility might be favorable for *Wolbachia*. However, through these manipulations, *Wolbachia* are able to increase their infection rates in the host population.

As opposed to their negative influence, many of the positive effects of *Wolbachia* on the host have only become known in the last fifteen years [8]. Cases of vitamin supplement, antiviral protection of the host by the *Wolbachia* symbiont, protection from some types of stresses and even an increase in host longevity have been demonstrated [8]. In short, *Wolbachia* can impact their host in many ways. It is possible that the beneficial effects on survival provided by *Wolbachia* almost balance out the negative effects associated with reproduction.

Relationships in the *Wolbachia*–host symbiotic system can be described as an intricate codependency and are a result of prolonged coevolution. *Wolbachia* genomes are incredibly diverse since they have accumulated a vast array of differences during evolution in various hosts. Isolation is a key factor in creating such diversity. As for the hosts, the connection between their genetic diversity and the presence of *Wolbachia* remains to be investigated. Since our study is focused on the effect of *Wolbachia* on *Drosophila melanogaster*, we will further describe the diversity of *Wolbachia* genotypes in this host in particular. The widely used classification of genotypes in *Wolbachia* infecting *D. melanogaster* was suggested by Riegler et al. [9] based on a number of genome structural variants, including genome rearrangements, copy number variants and IS5 transposable elements. Later, it was improved and now includes the following *Wolbachia* genotypes, which are divided into two groups: wMel, wMel2, wMel3 and wMel4 (the wMel group) and wMelCS and wMelCS2 (the wMelCS group).

Different *Wolbachia* strains were shown to have different effects on their host. For example, *Wolbachia* strains transferred to the same genetic background of *D. simulans* from different *Drosophila* species provided antiviral protection with different intensities [10]. Various *Wolbachia* genotypes transferred to the same genetic background of *D. melanogaster* have also demonstrated different effects on host hormonal status and survival under heat stress [11,12]. The effects on the host depend not only on the strain of *Wolbachia* but also on the host’s nuclear background [12,13,14]. Evidence suggests that survival depends on strong interactions between the *Wolbachia* infection and host genotype and also on the mating conditions.

We conducted a detailed investigation of both adverse and beneficial effects of *Wolbachia* on their host. Previously, we created a conplastic line in *D. melanogaster* with a nuclear genotype from the wild-type line, Bi90, and the cytoplasm of the line infected with the wMelCS genotype of *Wolbachia*. To obtain a line carrying the same nuclear genotype but a different strain of *Wolbachia*, cytoplasmic substitution was achieved after 20 generations of individual saturating crosses of females carrying cytoplasm with the *Wolbachia* strain of interest with males from the Bi90^T^ line. The Bi90^T^ line was obtained from the Bi90 wild-type *D. melanogaster* line treated with tetracycline for three generations prior to the start of crosses. Thus, the original infection in the Bi90 line was completely eliminated, and a new line infected with the wMelCS strain, Bi90^wMelCS^, was obtained. This model allowed us to discover the effect of the wMelCS genotype on the overall fitness and metabolism of juvenile hormones and dopamine [11,12].

Following these discoveries, we asked ourselves if the characteristics discovered in the Bi90^wMelCS^ line are distinctive from those of the lines infected with other isolates from the wMelCS genotype. Or have we stumbled upon a new *Wolbachia* strain with unique characteristics, similar to the previously described pathological strain, wMelPop [3], the difference being that our strain has a positive impact on the host? In order to answer this question, we created another model using carryover of the cytoplasm carrying *Wolbachia* with the wMelCS genotype from various isolates found in nature (the genotype was defined by the classification from Riegler et al. [9]) to the genetic background of the *D. melanogaster* line, Bi90^T^. Experiments on this new model showed that the increase in dopamine metabolism, body weight and lipid and glucose content is characteristic of flies infected with all strains under study, which belonged to the wMelCS genotype of *Wolbachia* [15,16]; however, only one line, carrying a strain of *Wolbachia* from line w153, was shown to increase the host’s resistance to heat stress [15]. Notably, this effect was also observed when the *Wolbachia* strain under study was transferred to the *D. melanogaster* wild-type line, Canton S [15]. The unique strain found was named wMelPlus.

We hypothesized that changes in the *Wolbachia* genome might cause the emergence of a stress-resistant phenotype in wMelPlus-infected flies. To better understand the underlying mechanisms of wMelPlus-induced host stress resistance, we performed whole-genome sequencing of the wMelPlus strain, which confirmed that it is, in fact, a new strain by revealing a large inversion that had not been described before [17]. The wMelCS^112^ and wMelPlus strains were found to share a very close genetic composition differing only by the above-mentioned chromosome inversion and by nine SNPs (seven in coding sequences, including synonymous and missense variants, and two in noncoding sequences). However, no genes that could explain the wMelPlus effect on the host were found among those altered by these SNPs. On the other hand, inversions are known to be able to strongly affect the bacterial phenotype, including colony morphology, antibiotic susceptibility, hemolytic activity and the expression of many genes [18]. The inversion in the wMelPlus genome included the well-known *Octomom* region, containing eight *Wolbachia* genes, the loss or amplification of which causes wMelPop over-proliferation [19,20]. However, only one copy of *Octomom* is present in the wMelPlus genome, just as in the wMelCS^112^ genome. Among the genes encoding products that excrete into the environment of the bacterial cell and, thereby, might be involved in the host–Wolbachia interactions [21], there are the protein-encoding gene, WMELPLUS_00535, and three copies of genes coding WsnRNA46—a long noncoding RNA in the inversion, unique for wMelPlus [17]. WMELPLUS_00535 presumably codes a transmembrane protein containing a type IV secretion system domain (TrbC/VIRB2 pilin). WsnRNA46 is known for employing the mechanism of RNA interference to upregulate a microtubular motor protein of the host, Dhc, which is important for *Wolbachia* transmission into *Drosophila* oocytes [22,23]. Though these genes might present some interest for disentangling the conundrum of the bacteria–host interaction, our main hypothesis is currently that this inversion causes dysregulation of the involved genes, which results in the disruption of the established genetic regulatory circuits and subsequent changes in *Drosophila* gene expression.

We can say that one inversion in a small genome is one giant leap for its host. However, we decided not to limit our examination of this symbiotic influence to the bacterium alone. After the whole genome of wMelPlus was obtained and analyzed, the next logical step was to obtain the transcriptome of flies infected with it.

The aim of our current work was to reveal the molecular mechanisms underlying the changes caused by wMelPlus *Wolbachia* in the host. Moreover, we searched for the differences in the changes in differential expression of genes in wMelPlus-infected flies and those infected with another strain, wMelCS^112^, which does not differ from other known variants of the wMelCS genotype, as it lacks the inversion that sets wMelPlus apart.

With this goal in mind, we decided to conduct transcriptomic analysis of three *D. melanogaster* lines: Bi90^wMelPlus^ and Bi90^wMelCS112^, carrying the wMelPlus and wMelCS^112^ strains of *Wolbachia*, respectively, and the uninfected Bi90^T^ line. It is of interest to compare the transcriptional response to the *Wolbachia* infection in the context of the interstrain variability.

At first, we considered using only the ovaries of flies, since it is known that these organs are rich in *Wolbachia*. However, it had been recently demonstrated that the effects of *Wolbachia* on the composition of mRNA transcripts of ovaries and early embryos of *D. melanogaster* are limited and variable [24,25]. The data obtained allowed Frantz et al. [25] to conclude that a *Wolbachia* infection explains only a small share of ovarian transcriptional variation compared with the variation among host lines. Furthermore, *Wolbachia* may affect genes that are expressed in other organs but not in the ovaries. It is also necessary to take into account batch effects known to be a huge contributing factor for fluctuations in the transcriptome caused by changing conditions [26]. Based on the aforementioned studies, we decided to investigate the whole female fly transcriptome, keeping in mind the importance of synchronizing insects used for the analysis in order to avoid transcriptional variation caused by factors other than the infection.

## 2. Results

### 2.1. Mapping and Quantification

Samples taken into analysis came from three Bi90^T^-based lines of flies: uninfected, infected with wMelPlus and infected with wMelCS^112^ *Wolbachia* strains. Four replicates for the uninfected line and three for each infected line were used to obtain pooled RNA for sequencing. Each replicate came from 20 whole, 10-day-old females. As a result, 20 RNA-seq libraries (forward and reverse reads) of 20 million reads were obtained with a length range of 75 to 76 bp. After subsequent quality control, 15.5 to 29.8 million reads per sample remained. Mapping results are presented in Table 1. Quantification was performed using featureCounts [27].

### 2.2. Analysis of Differentially Expressed Genes

Comparative analysis of the transcriptomes of uninfected flies and flies infected with two *Wolbachia* strains, wMelPlus and wMelCS^112^, showed significant differences in the expression levels of genes. The differences between the groups are complex and require detailed description, which is given below.

A total of 12,550 genes were identified and annotated. A total of 714 differentially expressed genes (DEGs) were found, including 493 DEGs from the comparison of the “Uninfected” and “Infected with wMelCS^112^” transcriptomes and 336 DEGs from the comparison of the “Uninfected” and “Infected with wMelPlus” transcriptomes. A total of 115 DEGs fell into both of these groups, which means they differentiate the *Wolbachia*-infected lines under study from the uninfected line.

**Figure 1:**
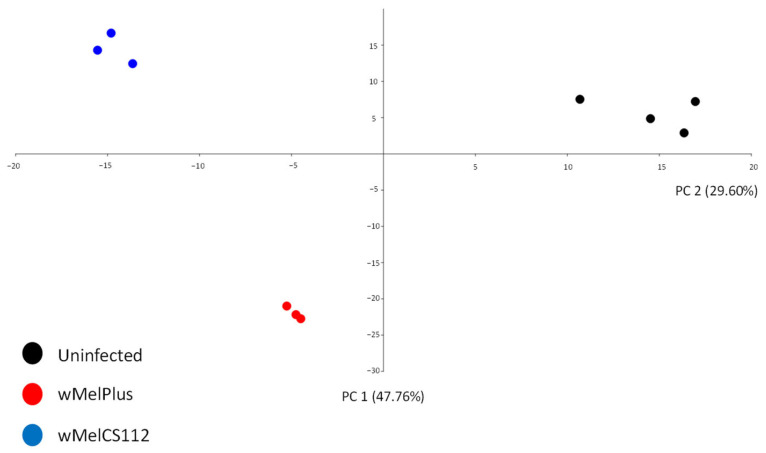
Principal component analysis of the RNA-seq data distribution. Components were calculated from a set of expression level values of DEGs (714 genes) for ten samples. The first and second components describe 77.3% of the variants. The uninfected Bi90^T^ line differs from the infected lines by PC2; the wMelPlus-infected line differs from the two other lines under study by PC1.

The estimated data distribution in the principal component analysis (PCA) matched the predicted one (Figure 1). The first and second components described 77.3% of the variants. The uninfected Bi90^T^ line differed from the infected lines by PC2; the wMelPlus-infected line differed from the two other lines under study by PC1.

**Figure 2:**
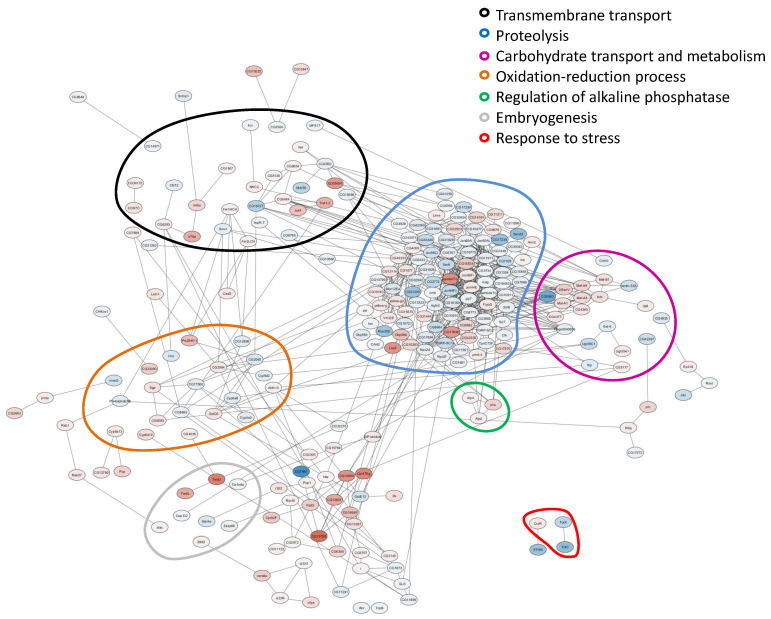
Gene networks for differentially expressed genes in wMelPlus-infected females compared to uninfected ones. The functional groups are enclosed in circles of different colors. The color of the node shows the direction and level of gene expression change in wMelPlus-infected females compared to uninfected ones. Color gradient from blue to red shows the expression level of genes relative to each other. Shades of blue indicate a decrease in expression; shades of red indicate an increase in expression. STRING was used to identify the core network of interactions at a confidence threshold of 0.4 (medium confidence).

**Figure 3:**
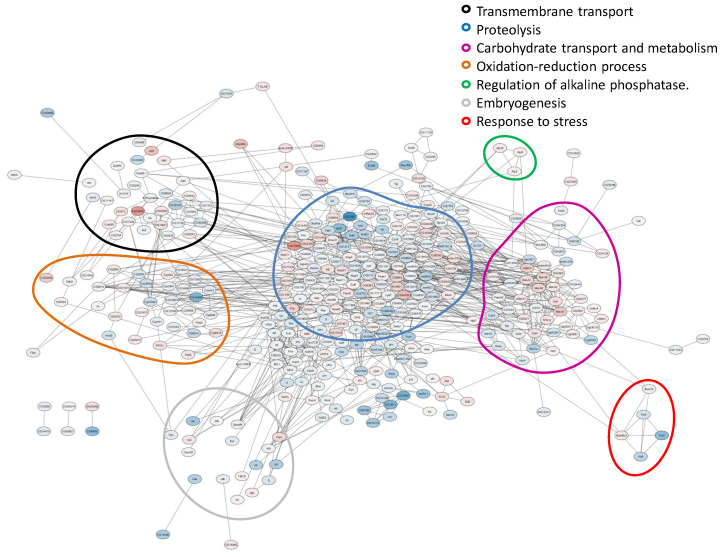
Gene networks for differentially expressed genes in wMelCS^112^-infected females compared to uninfected ones. The functional groups are enclosed in circles of different colors. The color of the node shows the direction and level of gene expression change in wMelCS^112^-infected females compared to uninfected ones. Color gradient from blue to red shows the expression level of genes relative to each other. Shades of blue indicate a decrease in expression; shades of red indicate an increase in expression. STRING was used to identify the core network of interactions at a confidence threshold of 0.4 (medium confidence).

For the DEGs found, gene networks were constructed using the STRING database. For the first pair, 368 nodes and 831 edges were obtained; for the second pair, 236 nodes and 615 edges were obtained. Based on the DEGs annotation, the following functional groups were designated: “Proteolysis”, “Carbohydrate transport and metabolism”, “Oxidation–reduction process”, “Embryogenesis”, “Transmembrane transport”, “Response to stress” and “Alkaline phosphatases” (Figure 2 and Figure 3).

**Figure 4:**
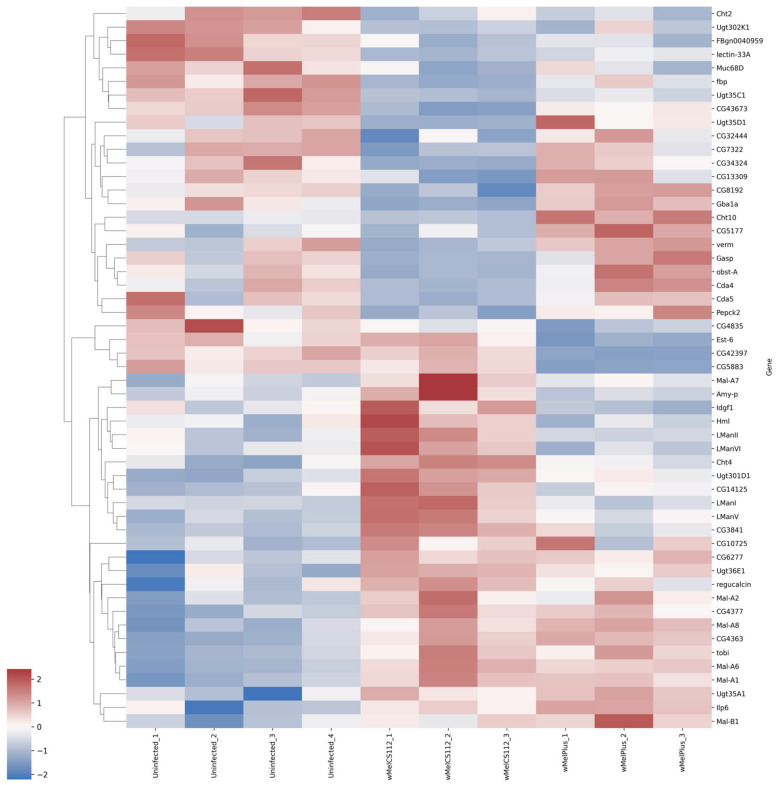
Heat map of differentially expressed genes belonging to the “Carbohydrate transport and metabolism” functional group in flies infected with wMelPlus and wMelCS^112^ *Wolbachia* strains compared to uninfected flies. Uninfected_1, Uninfected_2, Uninfected_3—gene expression of the uninfected *Drosophila melanogaster* line; wMelCS112_1, wMelCS112_2, wMelCS112_3—the *Drosophila melanogaster* line infected with wMelCS^112^; wMelPlus_1, wMelPlus_2, wMelPlus_3—the *Drosophila melanogaster* line infected with wMelPlus. Genes were clustered using the linkage method. The heat maps were generated using the Python3 package seaborn v0.13.0.

**Figure 5:**
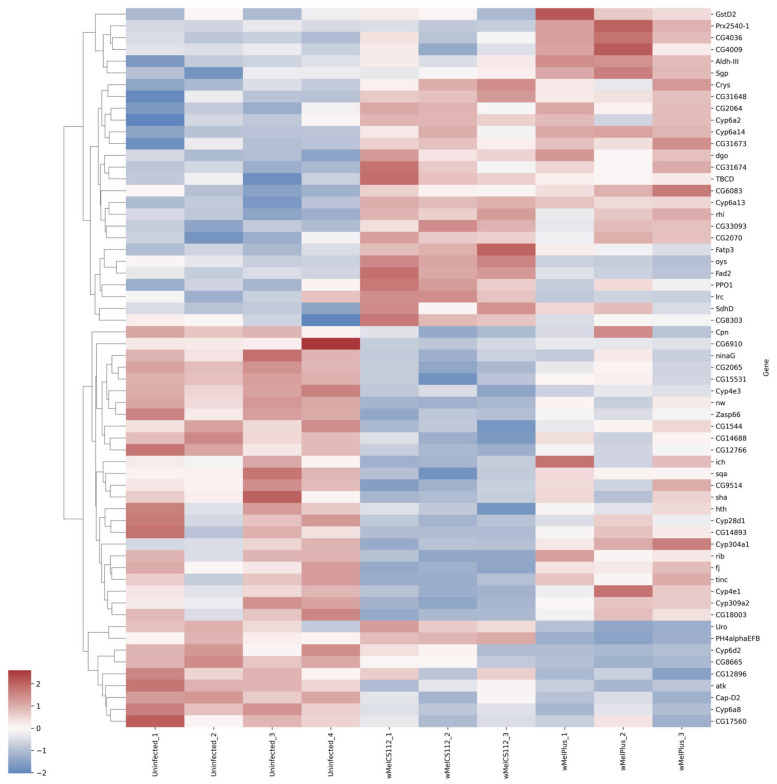
Heat map of differentially expressed genes belonging to the “Oxidation–reduction process” functional group in flies infected with wMelPlus and wMelCS^112^ *Wolbachia* strains compared to uninfected flies. Uninfected_1, Uninfected_2, Uninfected_3—gene expression of the uninfected *Drosophila melanogaster* line; wMelCS112_1, wMelCS112_2, wMelCS112_3—the *Drosophila melanogaster* line infected with wMelCS^112^; wMelPlus_1, wMelPlus_2, wMelPlus_3—the *Drosophila melanogaster* line infected with wMelPlus. Genes were clustered using the linkage method. The heat maps were generated using the Python3 package seaborn v0.13.0.

**Figure 6:**
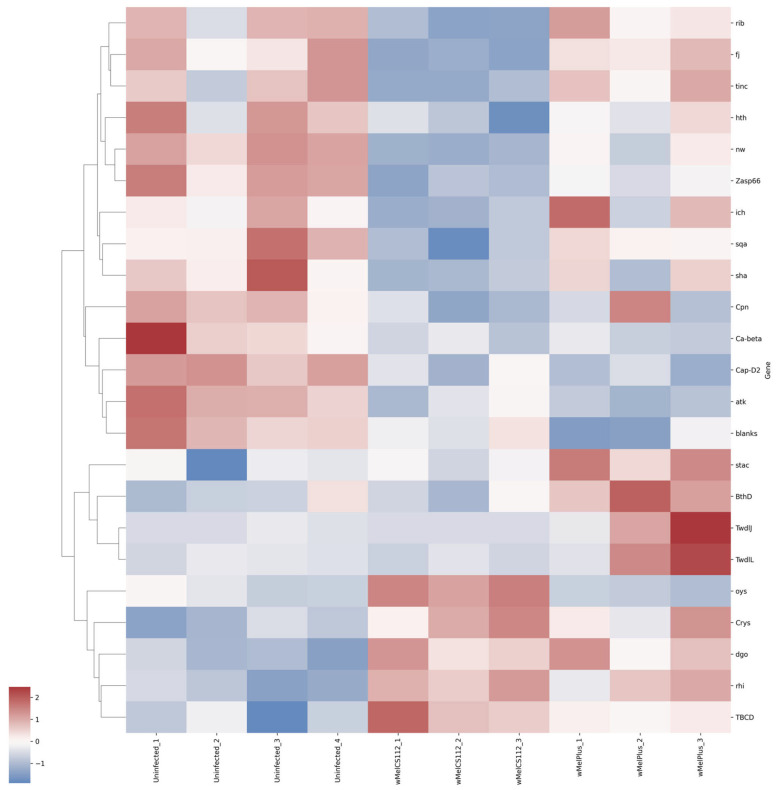
Heat map of differentially expressed genes belonging to the “Embryogenesis” functional group in flies infected with wMelPlus and wMelCS^112^ *Wolbachia* strains compared to uninfected flies. Uninfected_1, Uninfected_2, Uninfected_3—gene expression of the uninfected *Drosophila melanogaster* line; wMelCS112_1, wMelCS112_2, wMelCS112_3—the *Drosophila melanogaster* line infected with wMelCS^112^; wMelPlus_1, wMelPlus_2, wMelPlus_3—the *Drosophila melanogaster* line infected with wMelPlus. Genes were clustered using the linkage method. The heat maps were generated using the Python3 package seaborn v0.13.0.

**Figure 7:**
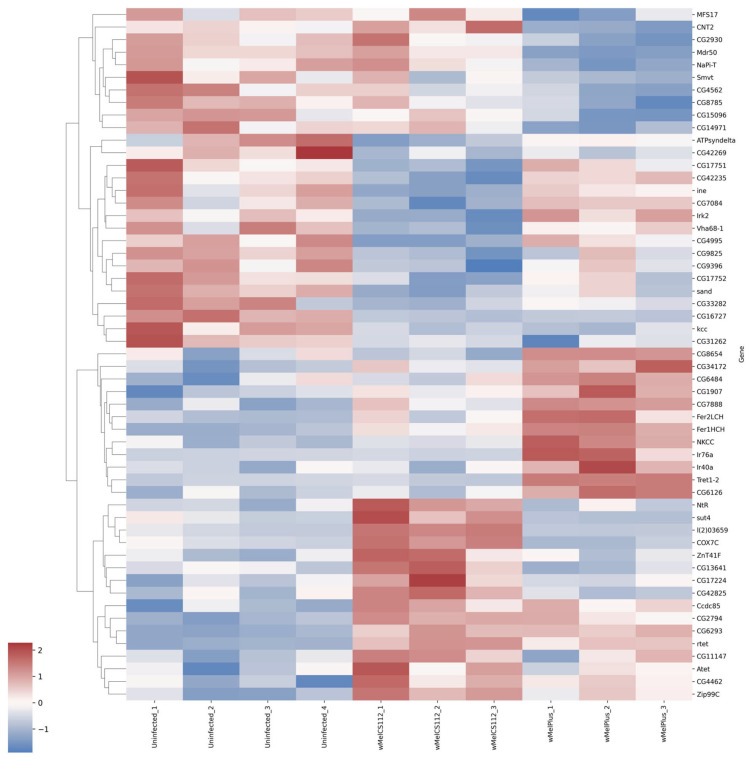
Heat map of differentially expressed genes belonging to the “Transmembrane transport” functional group in flies infected with wMelPlus and wMelCS^112^ *Wolbachia* strains compared to uninfected flies. Uninfected_1, Uninfected_2, Uninfected_3—gene expression of the uninfected *Drosophila melanogaster* line; wMelCS112_1, wMelCS112_2, wMelCS112_3—the *Drosophila melanogaster* line infected with wMelCS^112^; wMelPlus_1, wMelPlus_2, wMelPlus_3—the *Drosophila melanogaster* line infected with wMelPlus. Genes were clustered using the linkage method. The heat maps were generated using the Python3 package seaborn v0.13.0.

**Figure 8:**
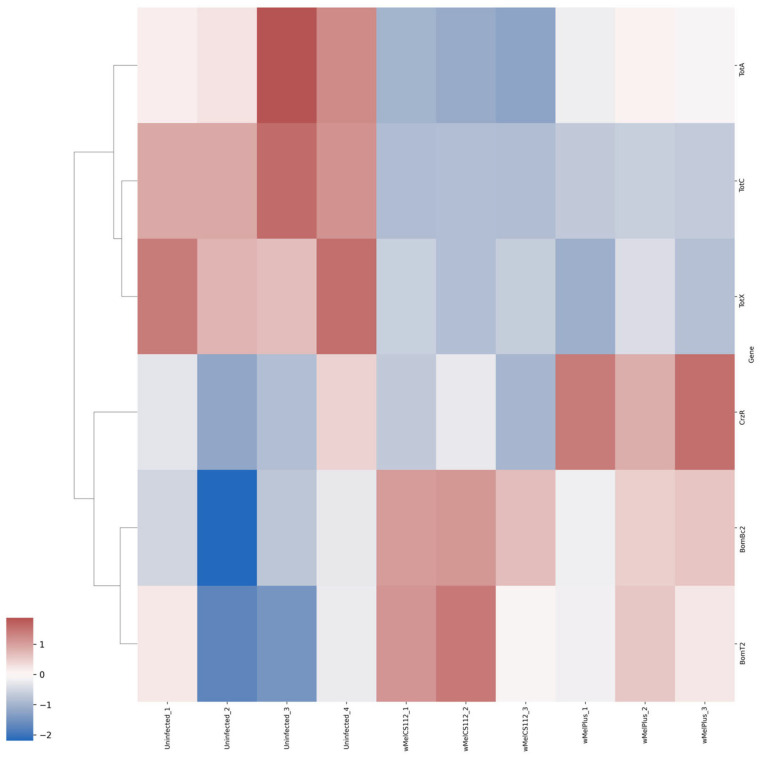
Heat map of differentially expressed genes belonging to the “Response to stress” functional group in flies infected with wMelPlus and wMelCS^112^ *Wolbachia* strains compared to uninfected flies. Uninfected_1, Uninfected_2, Uninfected_3—gene expression of the uninfected *Drosophila melanogaster* line; wMelCS112_1, wMelCS112_2, wMelCS112_3—the *Drosophila melanogaster* line infected with wMelCS^112^; wMelPlus_1, wMelPlus_2, wMelPlus_3—the *Drosophila melanogaster* line infected with wMelPlus. Genes were clustered using the linkage method. The heat maps were generated using the Python3 package seaborn v0.13.0.

**Figure 9:**
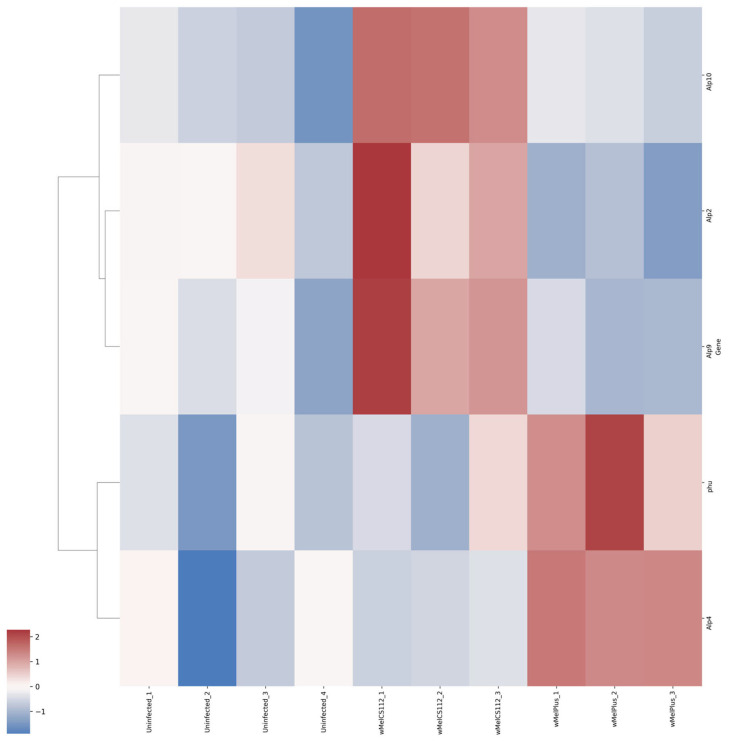
Heat map of differentially expressed genes belonging to the “Alkaline phosphatase” functional group in flies infected with wMelPlus and wMelCS^112^ *Wolbachia* strains compared to uninfected flies. Uninfected_1, Uninfected_2, Uninfected_3—gene expression of the uninfected *Drosophila melanogaster* line; wMelCS112_1, wMelCS112_2, wMelCS112_3—the *Drosophila melanogaster* line infected with wMelCS^112^; wMelPlus_1, wMelPlus_2, wMelPlus_3—the *Drosophila melanogaster* line infected with wMelPlus. Genes were clustered using the linkage method. The heat maps were generated using the Python3 package seaborn v0.13.0.

For both comparisons made, similar functional groups of DEGs were identified, but they contained different sets of genes, as can be seen from the heat maps constructed for them (Figure 4, Figure 5, Figure 6, Figure 7, Figure 8 and Figure 9 and Figure S1). It is important to emphasize that not all genes were included in the networks obtained: only 368 DEGs from the 493 DEGs in the comparison of the “Uninfected” and “Infected with wMelCS^112^” transcriptomes and 236 DEGs from the 336 DEGs in the comparison of the “Uninfected” and “Infected with wMelPlus” transcriptomes are connected to each other. However, the structure of the network is highly interconnected: a total of 831 edges are included for the “Uninfected–infected with wMelCS^112^” comparison and a total of 615 for the “Uninfected–infected with wMelPlus” comparison. The “Transmembrane transport”, “Carbohydrate transport and metabolism”, “Oxidation–reduction process” and “Embryogenesis” functional groups turned out to be strongly connected to the “Proteolysis” group. “Proteolysis” is the largest identified group, and “Alkaline phosphatase” is the smallest one. However, both of them strongly affect the host’s organism and vital functions.

We constructed heat maps for each designated functional group: “Proteolysis” (Figure S1), “Carbohydrate transport and metabolism” (Figure 4), “Oxidation–reduction process” (Figure 5), “Embryogenesis” (Figure 6), “Transmembrane transport” (Figure 7) and “Response to stress” (Figure 8). Each functional group contains both a set of genes that distinguish flies infected with either wMelCS^112^ or wMelPlus from the uninfected control and genes that distinguish flies infected with wMelCS^112^ from those infected with wMelPlus. Samples of the same fly lines show some variation in gene expression levels, but general tendencies toward an increase or decrease in expression in infected lines relative to the uninfected control remain. In the “Carbohydrate transport and metabolism” functional group, there are more genes with higher expression levels in wMelCS^112^-infected flies than in wMelPlus-infected ones. However, in all the other functional groups, the opposite tendency can be seen.

The same functional groups are identified in the data of RNA-seq of samples from another batch (Table S1, Figures S2 and S3) demonstrating the reproducibility of the results obtained on the first batch.

The analysis of DEGs involved in stress response revealed similar changes in the network related to two gene families, *Turandot* and *Bomanins,* in both infected lines compared to the uninfected one (Figure 8). What is unique for wMelPlus-infected females in relation to other groups under study is the increased level of expression of the corazonin receptor gene, *CrzR*.

One more functional group we distinguished in our analysis is the family of alkaline phosphatase genes. It could be seen that two representatives of this family, the *phu* and *Alp4* genes, are upregulated in females infected with the wMelPlus *Wolbachia* strain; and three others, the *Alp2, Alp9* and *Alp10* genes, are upregulated in females infected with the wMelCS^112^ strain (Figure 9).

**Figure 10:**
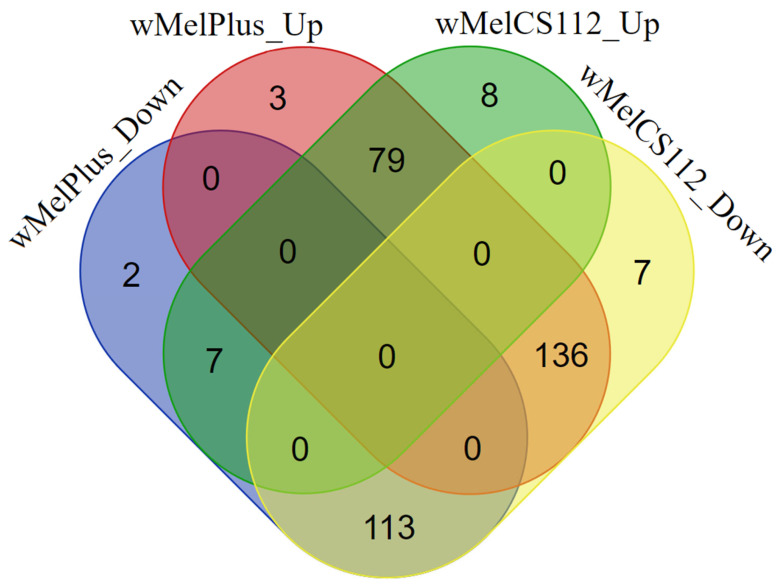
Euler diagram of four pairwise comparisons for DEGs included in the functional groups: upregulated DEGs in wMelPlus-infected flies vs. downregulated DEGs in wMelPlus-infected flies, upregulated DEGs in wMelCS^112^-infected flies vs. downregulated DEGs in wMelCS^112^-infected flies, upregulated DEGs in wMelPlus-infected flies vs. upregulated DEGs in wMelCS^112^-infected flies, downregulated DEGs in wMelPlus-infected flies vs. downregulated DEGs in wMelCS^112^-infected flies. The elliptical semi-transparent shapes indicate sets of DEGs: red ellipse for up-regulated DEGs in wMelPlus-infected flies, blue for down-regulated DEGs in wMelPlus-infected flies, green for up-regulated DEGs in wMelCS^112^-infected flies, yellow for down-regulated DEGs in wMelCS^112^-infected flies. The numbers indicate the quantity of genes belonging to one or more intersecting sets of DEGs.

Additionally, we evaluated relative expression levels of DEGs included in the functional groups. In total, 350 genes were identified as elements of these groups (Figure 10). A total of 218 genes had increased and 122 had decreased expression levels in the line infected with the wMelPlus strain relative to the uninfected control group. At the same time, a total of 94 genes had increased and 256 had decreased expression levels in the line infected with the wMelCS^112^ strain relative to the uninfected control group. In both infected lines, 192 genes had similar changes in the patterns of expression. More specifically, 79 genes were upregulated in both strains, while 113 were downregulated compared to the uninfected line. On the other hand, 143 genes changed their expression pattern. Seven genes increased their expression in flies infected with wMelCS^112^ and were decreased in flies infected with wMelPlus. At the same time, 136 genes increased their expression in the presence of wMelPlus and decreased their expression in the presence of wMelCS^112^.

Table 2, Table 3 and Table 4 show the distribution of annotated genes by major functional groups and the type of regulation. The tables also include those genes that are not represented in gene networks but are present in the heat maps. A clear distinction between the three lines under study can be seen. We would like to emphasize the existence of genes regulated similarly in both infected lines in all functional groups. These genes represent an overall response to the *Wolbachia* infection of the wMelCS group in *D. melanogaster* females.

Other genes in the functional groups not only differ in expression levels but also distinguish the “Uninfected—wMelCS^112^” pair from “Uninfected—wMelPlus”. All of the above indicates the importance of the selected functional groups.

### 2.3. qRT-PCR Analysis of Five Differentially Expressed Genes

**Figure 11:**
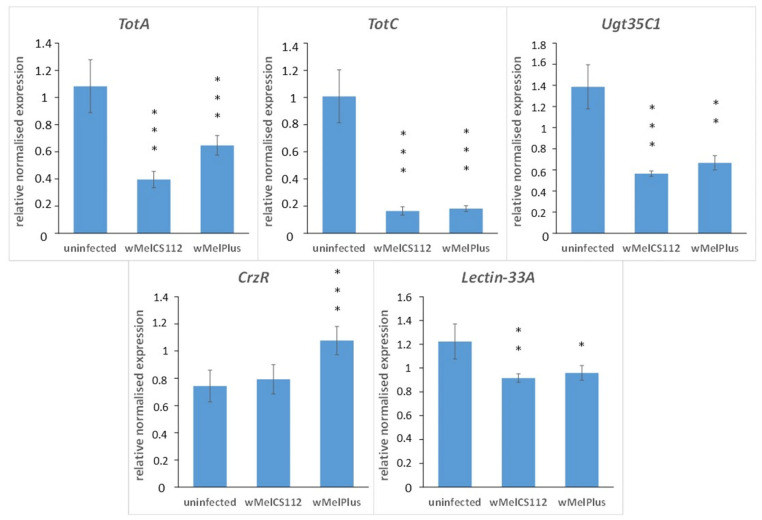
Relative gene expression in flies infected with wMelCS^112^ and wMelPlus *Wolbachia* strains (lines Bi90^wMelCS112^ and Bi90^wMelPlus^, respectively) and uninfected flies of line Bi90^T^ ± SEM. Asterisk indicates differences from the uninfected control. Three asterisks indicate *p* < 0.001, two asterisks—*p* < 0.01, one asterisk—*p* < 0.05 (*t*-test, applying Benjamini–Hochberg *p* adjustment to correct for multiple testing).

We validated the RNA-seq data using qRT-PCR in five DEGs: *TotA, TotC, Ugt35C1, CrzR* and *Lectin-33A* (Figure 11). The directionality and relative magnitude of change matched the transcriptome data in all cases. The expression of *TotA, TotC, Lectin-33A* and *Ugt35C1* was decreased in both infected strains (*p* < 0.001 for *TotA* and *TotC* in both Bi90^wMelCS112^ and Bi90^wMelPlus^; *p* < 0.01 for *Ugt35C1* in Bi90^wMelPlus^ and *Lectin-33A* in Bi90^wMelCS112^; *p* < 0.05 for *Lectin-33A* in Bi90^wMelPlus^). The expression level of *CrzR* in Bi90^wMelCS112^ did not differ from that in the uninfected Bi90^T^ line, but in Bi90^wMelPlus^, it was significantly higher than in Bi90^T^ (*p* < 0.001).

## 3. Discussion

Our findings showed that *Wolbachia* affects many biological processes in *D. melanogaster* females, for which the following functional groups can be designated: “Proteolysis”, “Carbohydrate transport and metabolism”, “Oxidation–reduction process”, “Embryogenesis”, “Transmembrane transport”, “Response to stress” and “Alkaline phosphatases”. Earlier, transcriptome analysis in *D. melanogaster* infected with *Wolbachia* was performed using virgin and mated females [28,29], embryos [24] and in dissected ovaries [25,30] and testes [29]. No significant differences were found in the mRNA composition between *Wolbachia*-infected embryos and uninfected ones [24], and the effect of *Wolbachia* on ovaries was rather limited: only 26 DEGs were identified in two batches 2 months apart [25]. It seems that most of the changes caused by *Wolbachia* in host expression start during later developmental stages. However, several gene ontology (GO) terms were found in another transcriptome analysis of *Wolbachia*-infected ovaries [30], including *metabolic process*, *development process*, and *response to stimulus*, which corresponds to our data on changes in the differential expression in the “Carbohydrate transport and metabolism” and “Embryogenesis” functional groups in Bi90^wMelPlus^ and Bi90^wMelCS112^ flies compared with the uninfected Bi90^T^ ones (see Figure 2 and Figure 3, Figures S2 and S3).

The results obtained in *D. melanogaster* females allowed the authors to relate them to the GO terms, *iron ion binding* and *oxidation–reduction process* [29], or create protein–protein interaction networks in STRING with the strongest interactions including “stress”, “ubiquitin”, “RNA binding and processing”, “transcription and translation” and “metabolism pathways” [28]. When comparing these results with our data, both similarities and differences were found. We also identified the “Oxidation–reduction process”, “Carbohydrate transport and metabolism” and “Response to stress” functional groups but did not find any groups connected with RNA synthesis and processing, protein synthesis or post-translational modification in the first batch (see Figure 2 and Figure 3); however, in the second batch, the “Ribosomal genes” functional group was revealed in wMelPlus-infected flies compared to the uninfected ones (see Figure S2). It seems that this functional group is rather unstable in its manifestations, which might be influenced by additional factors.

It Is worth noting that previous works on transcriptomic analysis of *D. melanogaster* lines carrying *Wolbachia* primarily focused on the wMel or wMel2 genotype [24,28,29,30]. To our knowledge, our research group was the first to conduct transcriptomic analysis of *D. melanogaster* lines infected with *Wolbachia* with a genotype from the wMelCS group. Since different *Wolbachia* strains were found to have different effects on the expression patterns of the host genes (see Figure 4, Table 2, Table 3 and Table 4), it could be supposed that the effect of the strains belonging to the wMel group of genotypes differed from that of the strains belonging to the wMelCS group. It is interesting that both strains having the most prominent effect on flies’ survival, wMelPop and wMelPlus, were found to be representatives of the wMelCS genotype [31], although the wMel genotype is much more widespread in nature compared to wMelCS [9,31,32,33]. The pathogenic wMelPop strain causes premature death of the *D. melanogaster* host [3,34,35], whereas infection with the wMelPlus strain promotes host survival under acute heat stress [11,15].

As to the results obtained in males or their testes, surprisingly, they also partially agree with our findings in female flies. Carbohydrate and lipid metabolism have been shown to be affected by *Wolbachia* infection both in adult testes [36] and whole males of *D. melanogaster* [37]. In testes, *Wolbachia* also affected differentially expressed genes involved in proteolysis [36], just as in Bi90^wMelPlus^ and Bi90^wMelCS112^ flies (see Figure 3 and Figure 4 and Figure S1). Notably, two genes responsible for the intracellular cholesterol transport (*Npc2f*, *Npc2d*) were upregulated in *Wolbachia*-infected testes [36], while they were downregulated in Bi90^wMelPlus^ and Bi90^wMelCS112^ females (see Figure S1). These contrasting results can probably be explained by sexual dimorphism: cholesterol is a precursor of 20-hydroxyecdysone, which, in female *Drosophila,* plays a major regulatory role in the control of oogenesis [38].

The following observations were also important to take into account. Both a transcriptional response to infection by any strain of *Wolbachia* and interstrain variability were observed simultaneously. The genotype of the symbiont largely determined the effect it had on the host insect (see Figure 4, Table 2, Table 3 and Table 4). Consequently, we can only assess a general response to the *Wolbachia* infection by comparing our results to other findings.

**Figure 12:**
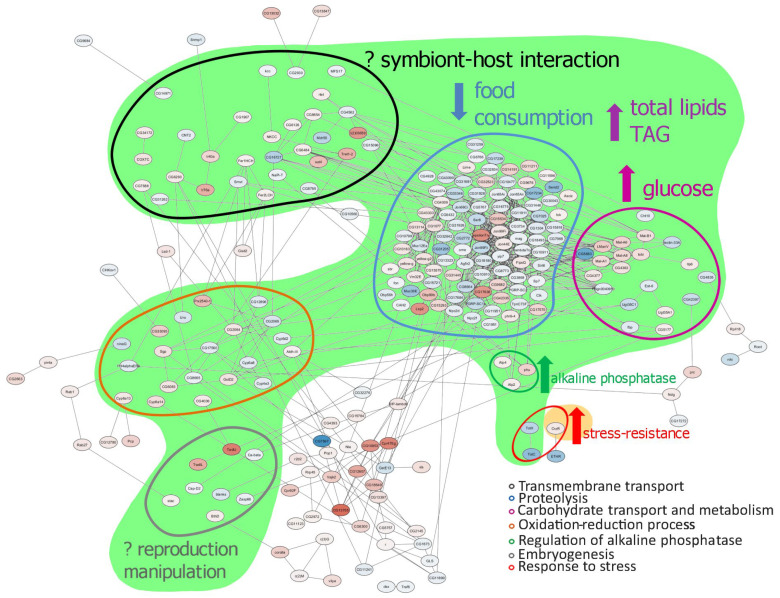
Scheme demonstrating the correlation between the changes in differential expression and the previously shown metabolic and fitness changes caused by *Wolbachia* [7,8,16]. Green region–changes caused by any *Wolbachia* strain, yellow region—changes caused by wMelPlus only. TAG—triglycerides. Upward pointing arrows indicate the increase of a parameter shown in *Wolbachia*-infected flies compared to uninfected ones, the downward pointing arrow indicates the decrease of a parameter shown in *Wolbachia*-infected flies compared to uninfected ones. The question marks indicate possible connection of changes in DEGs and *Wolbachia* effect on the host.

However, changes in metabolism could be considered common for *Wolbachia*-infected flies because they were demonstrated in several studies besides the present one [28,30,36,37]. This especially applies to carbohydrate transport and metabolism, the changes in which (see Figure 4, Table 2) correspond to increased glucose content in flies infected with different *Wolbachia* genotypes [16,36] (Figure 12).

It was shown earlier that *Wolbachia* depend on intermediate carbohydrates of the host to produce ATP and are able to manipulate the host’s energy metabolism in order to obtain these molecules [39]. In *D. melanogaster*, it was found that not only do infected flies have increased levels of glucose [16,36], but *Wolbachia* change the protein/carbohydrate ratio throughout the fly’s life [40]. This phenomenon can probably be explained by a competition for carbohydrates between *Wolbachia* and the host, which results in a decrease in the lifespan of infected flies under carbohydrate deficiency conditions.

The changes in proteolysis could also be considered common for flies infected with various *Wolbachia* strains, corresponding to changes in food consumption in the *D. melanogaster* lines infected with different *Wolbachia* genotypes and to increased starvation resistance demonstrated for the line infected with the wMelPlus strain [16,36]. It should be noted that the direction of these changes depends on sex: infected females demonstrate decreased appetite compared with uninfected ones [16], whereas infected males show increased food consumption [37]. Notably, changes in the activities of acid proteases, lipases and esterases were also found in *Wolbachia*-infected larvae of *Habrobracon hebetor* compared to uninfected ones [41].

As mentioned above, various *Wolbachia* strains altered gene expression in different ways (see Figure 10): the wMelCS^112^ strain mostly caused a decrease in the expression level of the genes belonging to the functional groups we identified in the present study, whereas wMelPlus mostly caused an increase in their expression. A total of 256 genes were downregulated in the wMelCS^112^-infected flies and only 122 in the wMelPlus-infected ones; 94 genes were upregulated in the wMelCS^112^-infected flies and 218 in wMelPlus-infected ones. The most notable genetic difference between the two strains was a large inversion. We find it most likely that such an inversion can significantly modify established genetic communications between the host and symbiont.

Perhaps the most importance aspect of our study were the changes in the expression levels of several stress-associated genes and genes encoding different homologs of alkaline phosphatase (Table 2, Figure 8 and Figure 9). The downregulation of the expression of three genes belonging to the *Turandot* family and upregulation of two *Bomanins* connected with them in both infected strains provide evidence for both the suppression of tolerance to long-lasting heat stress and activation of antimicrobial activity. *Turandot* genes are known to be part of a mechanism determining heat tolerance in *Drosophila* [42,43], and *Bomanin* genes have been shown to mediate immunity in *Drosophila* [44,45]. The latter agrees with data on the increased resistance of *Wolbachia*-infected insects to bacterial and fungal infections [8]. However, we believe that the most interesting result was the upregulation of the corazonin receptor gene, *CrzR*, in Bi90^wMelPlus^ flies, characterized by an increased resistance to acute heat stress [11,15]. Corazonin is a neuropeptide related to a vertebrate gonadotropin-releasing hormone and is known to alter stress physiology in *Drosophila*, mediating metabolic, osmotic and oxidative stress [46,47,48]. *CrzR* encodes a member of the Class A family of the G-protein-coupled receptor superfamily that transduces the corazonin signal in the salivary glands, the adult fat body and in seven pairs of corazonin neurons in the adult brain [48,49]. It was shown that the knockdown of *CrzR* in the adult fat body led to a compensatory increase in the corazonin peptide, which, in turn, resulted in an increase in stress resistance in *D. melanogaster* [48]. It seems that upregulation of *CrzR* in wMelPlus-infected flies produces a similar effect by increasing sensitivity to the corazonin signal.

Increased expression of the *phu* and *Alp4* genes in Bi90^wMelPlus^ flies and the *Alp2, Alp9* and *Alp10* genes in Bi90^wMelCS112^ flies correlated with increased alkaline phosphatase activity demonstrated in them [15] (Figure 12). Notably, the only *Alp* gene shown to be expressed in the adult brain, *Alp4* [50,51], was changed in Bi90^wMelPlus^ flies only, which supports our previous hypothesis [11] that increased stress resistance in wMelPlus-infected flies is connected with changes in dopamine metabolism. Dopamine is known to be produced in the insect brain and to participate in the neuroendocrine stress response [52,53]. It was shown that an elevated level of dopamine correlated with a decrease in resistance to acute heat stress [54]. It was also demonstrated that only one of the known mutations of the *Alp4* gene resulted in an abnormal stress response [55]. It seems that *Alp4* could encode an enzyme involved in dopamine synthesis, while the *Alp2*, *Alp9* and *Alp10* genes encode enzymes not related to dopamine metabolism and stress response. The increase in their activity shown in the flies infected with the *Wolbachia* strains of the MelCS group, other than wMelPlus [15], does not affect the stress resistance.

## 4. Materials and Methods

### 4.1. Drosophila Lines and Rearing

Ten-day-old females from three *D. melanogaster* lines were used in the study: inbred wild-type line Bi90^T^ treated with tetracycline for three generations to make it *Wolbachia*-free prior to the start of the experiments [11] and two conplastic lines, carrying the nuclear background of the Bi90^T^ line and cytoplasmic backgrounds with the *Wolbachia* strains, wMelCS^112^ or wMelPlus. Lines Bi90^CS112^ and Bi90^wMelPlus^ were produced by backcrossing *Wolbachia* donor females with Bi90^T^ males for 20 generations, as described earlier [15]. The corresponding *Wolbachia* donor lines, 3–112 and w153, were isolated from nature and characterized for any *Wolbachia* infections [15,32], which was identified as the wMelCS genotype, according to Riegler et al. [9]. However, sequencing and comparative genomic analysis of the wMelPlus and wMelCS^112^ genomes revealed a large chromosomal inversion in wMelPlus, distinguishing it from wMelCS^112^ and other known representatives of the wMelCS group of *Wolbachia* genotypes [17].

Flies were maintained on standard food (agar–agar, 7 g/L; corn grits, 50 g/L; dry yeast, 18 g/L; sugar, 40 g/L) in an MIR-554 incubator (Sanyo, Osaka, Japan) at 25 °C under a 12:12 h light–dark cycle; females were used for the experiments at the age of 10 days.

### 4.2. RNA Isolation, cDNA Library Construction and RNA Sequencing

Three (for the Bi90^wMelCS112^ and Bi90^wMelPlus^ lines) and four (for the Bi90^T^ line) independent biological replicates were obtained by RNA extraction from whole females (20 females per sample) collected on the same day for the first (main) batch. For the second (supplementary) batch, four (for the Bi90^wMelCS112^ line) and five (for the Bi90^T^ and Bi90^wMelPlus^ lines) independent biological replicates were obtained in a similar way. The total RNA was extracted using TRI Reagent (T-9424, Sigma, San Diego, CA, USA) according to the manufacturer’s instructions.

The total RNA quality was assessed using the Agilent Bioanalyzer 2100 system (Agilent Technologies, Santa Clara, CA, USA) with the RNA 6000 Nano Kit (Agilent Technologies, Santa Clara, CA, USA). For the RNA-seq library preparation, 1 μg of high-quality RNA per sample was used as the input material. mRNA fractions were isolated and barcoded. RNA-seq libraries for the Illumina system were constructed using the TruSeq Stranded mRNA Library Prep Kit (Illumina, San Diego, CA, USA) according to the manufacturer’s instructions. The quantity and quality of the libraries were assessed using the Agilent Bioanalyzer 2100 system (Agilent Technologies, Santa Clara, CA, USA) and the DNA High Sensitivity Kit (Agilent Technologies, Santa Clara, CA, USA). The libraries were sequenced using the Illumina NextSeq 550 with 75-bp read length and a sequencing depth of 20 million reads per library.

### 4.3. Transcriptome Assembly and Quantification

Quality control was performed using FastQC v0.11.5 [56]. Adapters and primers were removed in Trimmomatic v.0.3 [57] using ILLUMINACLIP parameters (with built-in SE-library of TruSeq3-SE index adapters). ILLUMINACLIP:TruSeq3-SE.fa:2:30:10 LEADING:3 TRAILING:3 SLIDINGWINDOW:3:15 MINLEN:75 CROP:75 parameters were used to remove short and low-quality reads. The contig assembly was performed using Hisat2 [58] with the default settings on merged libraries with the annotated genome (GCF_000001215.4). A sorted BAM file was made using SamTools [59]. The quantification was performed using the featureCounts program [27]. The supplementary batch was mapped and quantified using the kallisto algorithm [60].

### 4.4. Gene Annotation and Analysis of Differential Expression

Annotation of genes and biochemical pathways was done using ShinyGOv77 [61] and the Flybase database [62]. Differential expression analysis was performed with the use of the edgeR v. 4.3 software package [63]. Results with an FDR parameter value of less than 0.05 were selected as differentially expressed genes. All DEGs, regardless of the log2 fold change value, remained included to maximize information retention. Log2 fold changes were used in all analyses and figures, except heat maps; counts per million (CPM) were used there instead.

### 4.5. Quantitative Real-Time Polymerase Chain Reaction

For quantitative real-time polymerase chain reaction (qRT-PCR) analysis, the RNA extraction procedure was identical to that for RNA sequencing. The synthesis of cDNA was carried out using the ABScript III RT Master Mix for qPCR with gDNA Remover #RK20429 (Abclonal Technology, Woburn, MA, USA). The expression of genes under study was analyzed on the CFX96 (Bio-Rad, Hercules, CO, USA) with qRT-PCR using three replicates for every sample. The qPCR mix contained R-402 c SYBR-Green I (Syntol, Moscow, Russia). Each reaction was performed with three biological replicates. The expression was quantified using the relative 2^−∆∆Ct^ method [64,65] with internal control primers specific for *β-Tubulin* and *Actin 5C* [65]. Primer sequences are given in Table 5.

### 4.6. Statistical Analysis

Principal components were calculated from a set of expression level values of DEGs (714 genes) for ten samples. The expression profiles were filtered to exclude null values. Next, for each gene, the expression profile was centered and normalized. Any excess of the number of traits over the number of objects was not an obstacle; the calculation of principal components can be done through an SVD transformation of the object–trait matrix at any ratios for the number of objects and traits. The analysis was performed by using the Past package v4.13 [66].

When conducting the qRT-PCR, three biological replicates were used, each of which was obtained from three technical ones. The CFX96 System (Bio-Rad, USA) reports only the mean of three technical values and the mean error, so it is impossible to check normality, use nonparametric tests or even apply bootstrap. However, from this information, it is easy to obtain the sum of the squares of the three technical values. This was enough to calculate both the overall mean and its error. The number of technical values for each overall mean was 9. When calculating the Student’s *t*-test, we obtained 2 × 9 − 2 = 16 degrees of freedom. It is known that the criteria of significance, such as in the Student’s *t*-test, are resistant to deviations from normality [67] due to the distribution of means approaching normality with an increasing sample size. Since a total of 10 comparisons were made, the Benjamini–Hochberg correction was additionally calculated for the *p*-value to compare with three standard significance levels [68].

## 5. Conclusions

In this study, we aimed to investigate the following aspects of host–bacteria interaction: the effects of the presence of the cytoplasmically-transmitted bacteria *Wolbachia* on the insect host’s gene expression patterns and a possible correlation of these effects with previously described changes in host fitness and metabolism caused by *Wolbachia*. We employed a transcriptomics approach to investigate how *Wolbachia* affects differential expression of genes in *D. melanogaster* females and whether a large chromosomal inversion in the bacterial genome influences the manner in which the host’s gene expression is altered by the *Wolbachia* infection. Our analyses revealed 493 DEGs that exhibit significant changes in expression in the flies infected with the wMelCS^112^ *Wolbachia* strain, which is a typical representative of the wMelCS group of genotypes, and 336 DEGs that exhibit significant changes in expression in the flies infected with the wMelPlus *Wolbachia* strain, which differed from other representatives of the wMelCS group by an inversion. A total of 368 DEGs and 236 DEGs from these two groups, respectively, were linked into gene networks, in which the following functional groups were designated based on DEGs annotations: “Proteolysis”, “Carbohydrate transport and metabolism”, “Oxidation–reduction process”, “Embryogenesis”, “Transmembrane transport”, “Response to stress” and “Alkaline phosphatases”. Thus, we demonstrated that the *Wolbachia* infection contributed to differential expression of host genes in a significant way. We also found that the list of differentially expressed genes differed between the wMelPlus-infected and wMelCS^112^-infected females, which indicates the important role of the bacterial genotype in *Wolbachia*–host interactions. This suggestion is also supported by the fact that the wMelPlus infection results in more than twice the number of upregulated DEGs and half the number of downregulated DEGs compared to the wMelCS^112^ infection. Notably, the only stress-related gene, the expression of which was found to be increased in stress-resistant flies (infected with wMelPlus) compared to control flies (uninfected and infected with the wMelSC^112^ strain), was the corazonin receptor gene, *CrzR*. In the future, we plan to research the role of the *CrzR* gene in providing resistance to factors such as heat stress in *Wolbachia*-infected flies.

## Figures and Tables

**Table 1:** Number of RNA-seq reads mapped to the *Drosophila melanogaster* genome per sample.

Sample	All Reads (Forward + Reverse)	Assigned	Unassigned
Uninfected_1	29,820,844	19,513,634	10,307,210
Uninfected_2	19,028,518	15,259,347	3,769,171
Uninfected_3	15,927,166	13,118,210	2,808,956
Uninfected_4	23,642,114	18,570,393	5,071,721
wMelPlus_1	17,379,117	14,243,016	3,136,101
wMelPlus_2	15,325,069	12,796,552	2,528,517
wMelPlus_3	24,559,750	18,973,636	5,586,114
wMelCS112_1	22,813,214	16,048,571	6,764,643
wMelCS112_2	18,300,298	15,087,798	3,212,500
wMelCS112_3	22,668,148	19,149,909	3,518,239

**Table 2:** Comparison between females of two *Drosophila melanogaster* lines infected with wMelCS^112^ and wMelPlus *Wolbachia* strains by expression levels of annotated DEGs included in functional groups, in relation to the uninfected control group.

Functional Group	Relative Expression Level	Strain	Gene
Oxidation–reduction process	Upregulated	Both	*rhi, Cyp6a13, Cyp6a14, TBCD, Crys, dgo*
		wMelCS^112^	*PPO1, Irc, Fatp3, oys, Fad2*
		wMelPlus	*Prx2540-1*
	Downregulated	Both	*Cyp6d2, atk, Cap-D2, Cyp6a8, Cpn*
		wMelCS^112^	*ich, sha, sqa, nw, Zasp66, ninaG, Cyp309a2, rib, fj, tinc, hth*
		wMelPlus	*Uro, PH4alphaEFB*
Carbohydrate metabolism and transportation	Upregulated	Both	*Mal-A2, Mal-A6, Mal-A1, Ugt35A1, Ugt36E1*
		wMelCS^112^	*Mal-A7, Amy-p, Hml, LManI, LManII, LManV, LManVI, Cht4, Ugt301D1*
		wMelPlus	*Cht10, Gba1a*
	Downregulated	Both	*Cht2, lectin-33A, Ugt35C1, Ugt302K1, fbp, Est-6*
Transmembrane transport	Upregulated	Both	*rtet, Atet, Zip99C*
		wMelCS^112^	*Fer1HCH, Ir40a, Tret1-2, NKCC, Ir76a*
		wMelPlus	*NtR, sut4, l(2)03659, COX7C*
	Downregulated	Both	*kcc*
		wMelCS^112^	*Irk2, ine, sand*
		wMelPlus	*Mdr50, NaPi-T, Smvt*
Alkaline phosphatases	Upregulated	wMelCS^112^	*Alp2, Alp9, Alp10*
		wMelPlus	*Phu, Alp4*
Response to stress	Upregulated	Both	*BomBC2, BomT2*
		wMelPlus	*CrzR*
	Downregulated	Both	*TotA, TotC, TotX*

**Table 3:** Comparison between females of two *Drosophila melanogaster* lines infected with wMelCS^112^ and wMelPlus Wolbachia strains by expression levels of annotated DEGs included in the “Embryogenesis” functional group in relation to the uninfected control group.

Relative Expression Level	Strain	Gene	Subgroup
Upregulated	Both	*rin, TBCD, dgo, gkt*	Establishment or maintenance of cell polarity
		*vls, rhi, rin, rasp, r2d2, sima*	Oogenesis
		*Crys*	Structural constituent of eye lens
		*rhi, rin, rasp, r2d2, RhoGEF64C, dgo, Hph*	Epithelium development
	wMelCS^112^	*Egr*	Cytokine receptor binding
		*lectin-37Da, lectin-37Db*	Galactose binding
		*oys*	Lysophospholipid acyltransferase activity
		*Invadolysin*	Metalloendopeptidase activity
		*Crim, egr*	Other molecular function
	wMelPlus	*TwdlL, TwdlJ*	Structural constituent of chitin-based cuticle
		*Loxl1*	Cargo receptor activity
		*Lsp2*	Nutrient reservoir activity
		*stac*	Cation binding
Downregulated	Both	*ft, sns, stan, plum, otk2*	Cell adhesion molecule binding
		*Idgf3, Idgf2*	Chitinase activity
		*D, hth, nerfin-1, Oli, ab*	DNA-binding transcription factor activity
		*blanks*	Double-stranded RNA adenosine deaminase activity
		*sqa*	Myosin light chain kinase activity
		*spz6*	Signaling receptor binding
		*slo*	Calcium activated cation channel activity
		*Hdc*	Carboxylyase activity
		*Zasp66*	Actinin binding
		*Ca-β, Cap-D2, beat-VII, atk, wkd, MFS14, cv-d, neo, sha, mas, nw*	Other molecular function
	wMelCS^112^	*Irk2*	Inward rectifier potassium channel activity
		*bves*	CAMP binding
		*ine*	Small molecule sensor activity
		*pnt, ich*	DNA-binding transcription activator activity
		*Tig*	Integrin binding
		*Lectin-galC1*	Galactose binding
		*fj*	Wnt-protein binding

**Table 4:** Comparison between females of two *Drosophila melanogaster* lines infected with wMelCS^112^ and wMelPlus Wolbachia strains by expression levels of annotated DEGs included in the “Proteolysis” functional group in relation to the uninfected control group.

Relative Expression Level	Strain	Gene	Subgroup
Upregulated	Both	*phr6-4*	Deoxyribodipyrimidine photolyase activity
		*Vm32E*	Structural constituent of vitelline membrane
		*sbr*	Protein N-terminus binding
		*lok*	Tau-protein kinase activity
		*Bace*	Aspartic-type endopeptidase activity
		*rasp*	Palmitoyltransferase activity
		*Jon44E*	Endopeptidase activity
		*tobi*	Hydrolase activity, hydrolyzing O-glycosyl compounds
		*Obp99b*	Odorant binding
		*Vm32E*	Other molecular function
	wMelCS^112^	*Nepl21, Nep6*	Metalloendopeptidase activity
		*Amy-p*	Alpha-amylase activity
		*egr*	Tumor necrosis factor receptor binding
		*Agpat2*	1-acylglycerol-3-phosphate O-acyltransferase activity
		*Myd88*	Toll binding
		*lectin-37Da*	Galactose binding
		*Phae1*	Catalytic activity, acting on a protein
		*DnaJ-H*	Unfolded protein binding
		*NimC1, Cul6, Agpat2, BomBc1, Hml*	Other molecular function
	wMelPlus	*Gasp, obst-A, Cda4, verm*	Chitin binding
		*epsilonTry, Jon66Ci, Jon66Cii, Jon99Fi, Nepl11, Psa*	Peptidase activity
		*Pdk, Gasp, obst-A, bves, Cda4, verm, hll*	Carbohydrate derivative binding
		*Lsp2*	Nutrient reservoir activity
		*yellow-g2, yellow-g*	Isomerase activity
Down regulated	Both	*neo, Muc12Ea, Muc30E, Muc68D,*	Extracellular matrix structural constituent
		*LysP, mas, Nepl3, Jhedup, Jon65Aii, mag, Sirt6, yip7, PGRP-SC2, PGRP-SC1a, Cda5, Send2*	Hydrolase activity
		*Npc2d, Npc2f*	Steroid binding
		*Lectin-galC1*	Galactose binding
		*tn*	Translation repressor activity
		*Cp16, Clk, Ag5r2, Obp56h*	Other molecular function
	wMelCS^112^	*Pdk, Gasp, Vajk1, obst-A, bves, Cda4, verm, hll*	Carbohydrate derivative binding
		*spz6*	Toll binding
		*Jon66Cii, Nepl11*	Endopeptidase activity
		*Obp83a, TpnC73F, ImpE2*	Other molecular function
	wMelPlus	*Ser8, Jon65Ai, Jon99Fii, Sp7, lambdaTry, ome*	Serine-type peptidase activity
		*CAH2*	Carbonate dehydratase activity
		*fon*	Other molecular function

**Table 5:** Primer sequences used for qRT-PCR.

Gene Name	Forward	Reverse
*TotA*	AATTCTTCAACTGCTCTTATGTGCT	CAGCAATTCTAAGGTTGTCAGC
*TotC*	CAGTTTGTCTTAAACCAGTGCTC	CAGATTCCCTTTCCTCGTCAG
*Ugt35C*	CAGACGAGATGATGAGTTTGCT	ACCAGATCGAAAGTTTCTCCC
*CrzR*	AATCCGGACAAAAGGCTGGG	AGGTGGAAGGCACCGTAGAT
*lectin-33A*	GAGTCGGAAACAAGTGCTACC	CGTGGTTGTGAGGAGTTTGTC
*β-Tubulin*	TGTCGCGTGTGAAACACTTC	AGCAGGCGTTTCCAATCTG
*Actin 5C*	GCGCCCTTACTCTTTCACCA	ATGTCACGGACGATTTCACG

## Data Availability

Data have been deposited in the GEO database; the accession numbers have not yet been obtained and will be provided during the review.

## Supplementary Materials

The following supporting information can be downloaded at: https://www.mdpi.com/article/10.3390/ijms242417411/s1.
