# Supplementary material for: Drosophila melanogaster Transcriptome Response to Different Wolbachia Strains
Source: Int J Mol Sci. 2023 Dec 12;24(24):17411. doi: 10.3390/ijms242417411 (PMC10743526; doi:10.3390/ijms242417411)

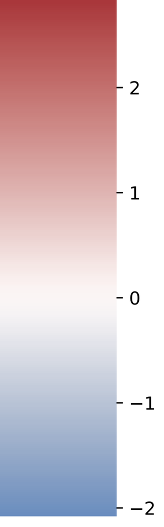

Figure S1. Heat map of differentially expressed genes belonging to the “Proteolysis” functional group in flies infected with wMelPlus and wMelCS112 Wolbachia strains compared to uninfected flies. Uninfected_1, Uninfected_2, Uninfected_3 – gene expression of the uninfected Drosophila melanogaster line; wMelCS112_1, wMelCS112_2, wMelCS112_3 – the Drosophila melanogaster line infected with wMelCS112; wMelPlus_1, wMelPlus_2, wMelPlus_3 – the Drosophila melanogaster line infected with wMelPlus. Genes were clustered using the linkage method. The heat maps were generated using the Python3 package seaborn.

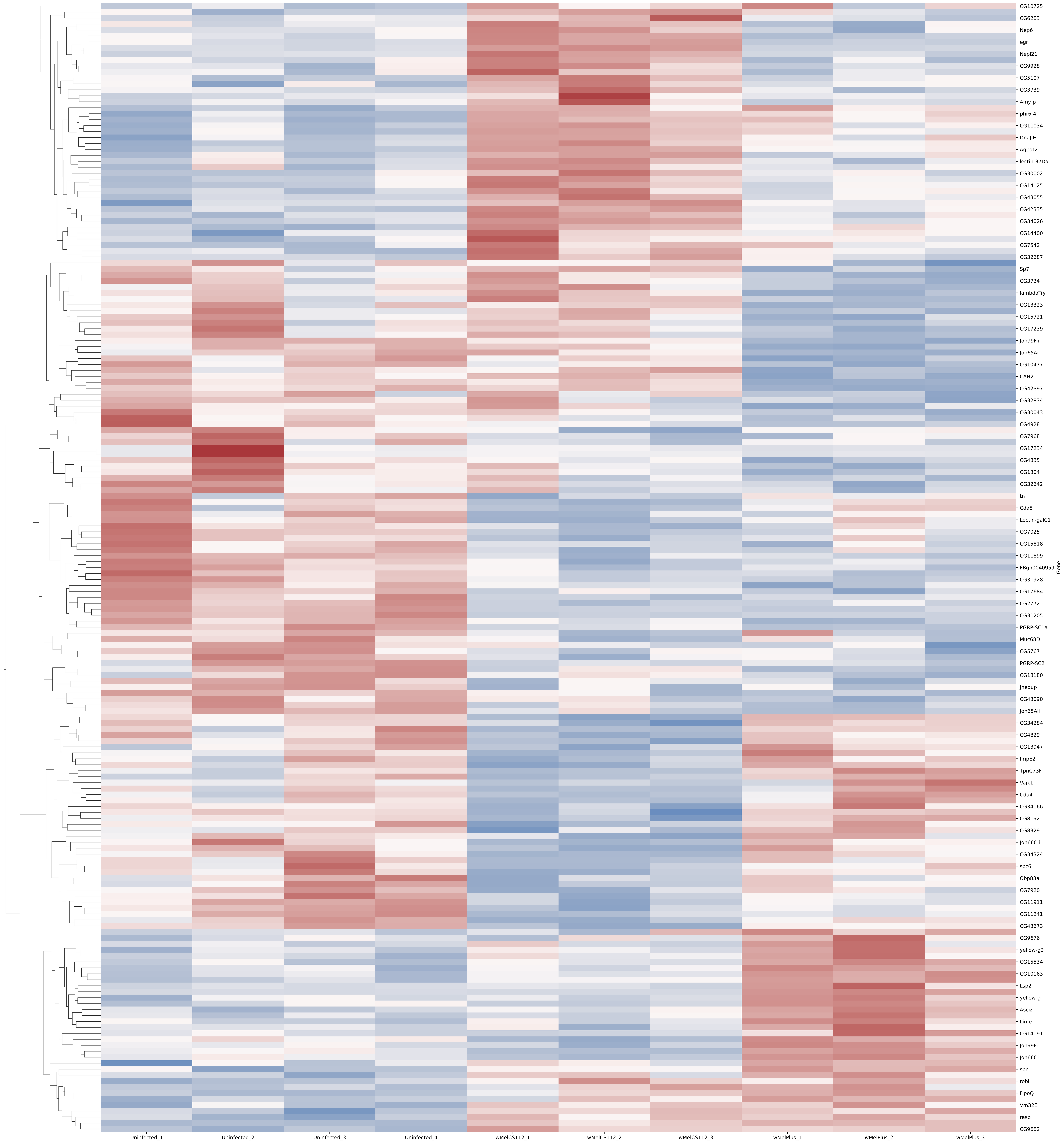

Supplement: Supplementary file 1 [file ijms-24-17411-s001.zip › Figure S1.pdf]
