# Supplementary material for: Drosophila melanogaster Transcriptome Response to Different Wolbachia Strains
Source: Int J Mol Sci. 2023 Dec 12;24(24):17411. doi: 10.3390/ijms242417411 (PMC10743526; doi:10.3390/ijms242417411)
Supplement: Supplementary file 1 [file ijms-24-17411-s001.zip › Figure S3.pdf]

Uninfected/wMelCS112 2

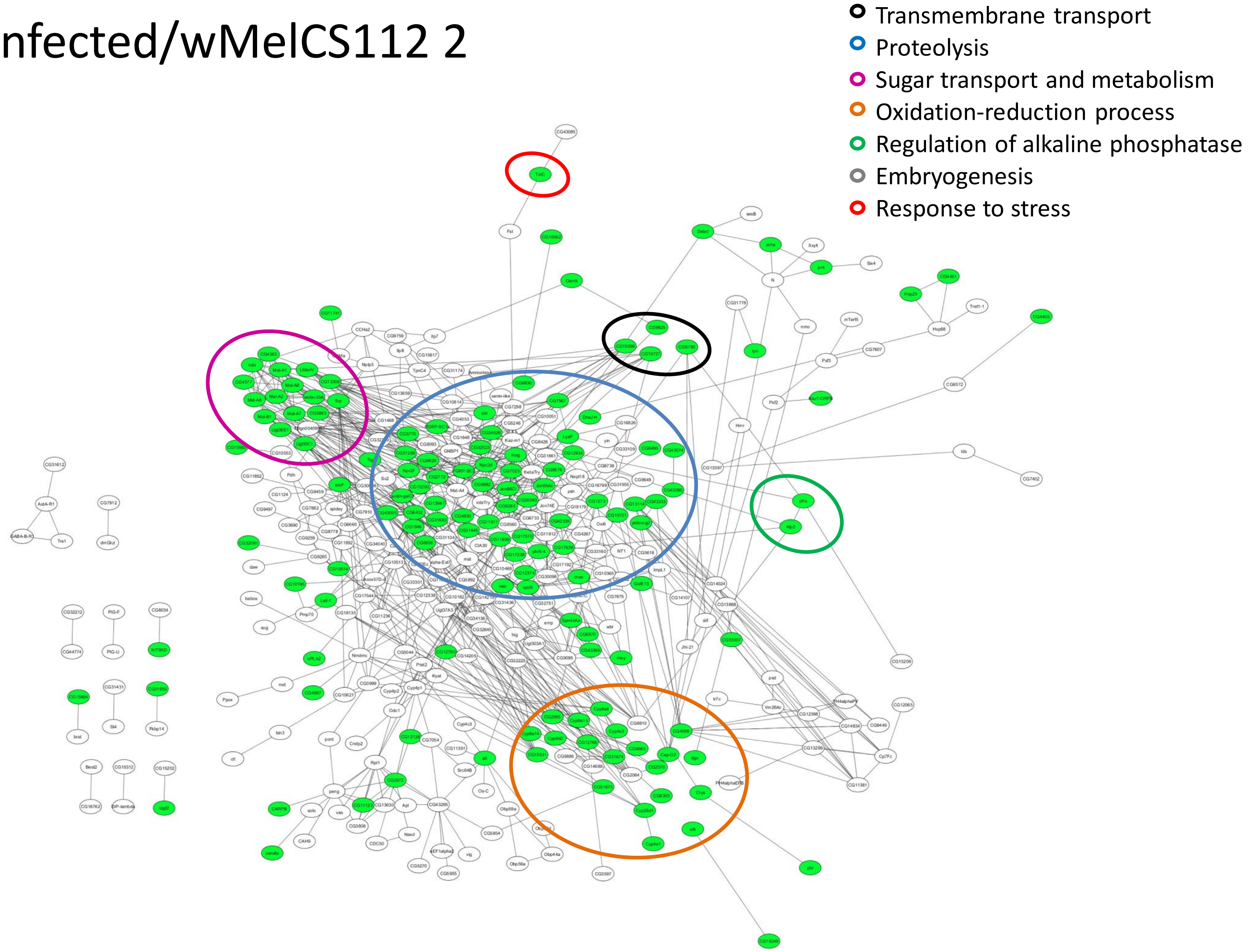

Figure S3. Gene networks for differentially expressed genes in wMelCS^{112}-infected females compared to uninfected ones (second batch). The functional groups are enclosed in circles of different colors. The green color of the node shows shows genes, the expression of which was found to change in wMelCS^{112}-infected females compared to uninfected ones in the first batch.
