# Supplementary material for: Drosophila melanogaster Transcriptome Response to Different Wolbachia Strains
Source: Int J Mol Sci. 2023 Dec 12;24(24):17411. doi: 10.3390/ijms242417411 (PMC10743526; doi:10.3390/ijms242417411)
Supplement: Supplementary file 1 [file ijms-24-17411-s001.zip › Table S1.pdf]

Table S1. Number of RNA-Seq reads mapped to the *Drosophila melanogaster* genome per sample in the second batch.

Sample	All reads (forward + reverse)	Assigned	Unassigned
Uninfected_1	28471619	28001382	470237
Uninfected_2	28930877	28552283	378594
Uninfected_3	27038238	26615511	422727
Uninfected_4	26551383	26131431	419952
Uninfected_5	24876716	24486820	389896
wMelPlus_1	20895748	20571335	324413
wMelPlus_2	17600250	17316110	284140
wMelPlus_3	22547845	22191248	356597
wMelPlus_4	24449981	24075216	374765
wMelPlus_5	21501131	21150899	350232
wMelCS112_1	36077645	35588928	488717
wMelCS112_2	22140664	21769083	371581
wMelCS112_3	28841480	28405479	436001
wMelCS112_4	22998196	22616043	382153
